# The Role of Impulsivity and Reward Deficiency in “Liking” and “Wanting” of Potentially Problematic Behaviors and Substance Uses

**DOI:** 10.3389/fpsyt.2022.820836

**Published:** 2022-04-25

**Authors:** Domonkos File, Beáta Bőthe, Bálint File, Zsolt Demetrovics

**Affiliations:** ^1^Institute of Psychology, ELTE Eötvös Loránd University, Budapest, Hungary; ^2^Department of Psychology, Université de Montréal, Montreal, QC, Canada; ^3^Wigner Research Centre for Physics, Budapest, Hungary; ^4^Centre of Excellence in Responsible Gaming, University of Gibraltar, Gibraltar, Gibraltar

**Keywords:** incentive sensitization, impulsivity, reward deficiency, problem behavior, substance misuse

## Abstract

A few studies have examined the changes in substance- and behavior-related “wanting” and “liking” of human subjects, the key properties of Incentive Sensitization Theory (IST). The aim of this study was to examine the dissociation between “wanting” and “liking” as a function of usage frequency, intensity, and subjective severity in individuals across four substances (alcohol, nicotine, cannabis, and other drugs) and ten behaviors (gambling, overeating, gaming, pornography use, sex, social media use, Internet use, TV-series watching, shopping, and work). Also, the potential roles of impulsivity and reward deficiency were investigated in “wanting,” “liking,” and wellbeing. The sex differences between “wanting” and “liking” were also examined. Based on our findings using structural equation modeling with 749 participants (503 women, *M*_*age*_ = 35.7 years, *SD* = 11.84), who completed self-report questionnaires, “wanting” increased with the severity, frequency, and intensity of potentially problematic use, while “liking” did not change. Impulsivity positively predicted “wanting,” and “wanting” positively predicted problem uses/behaviors. Reward deficiency positively predicted problem uses/behaviors, and both impulsivity and problem uses/behaviors negatively predicted wellbeing. Finally, women showed higher levels of “wanting,” compared to men. These findings demonstrate the potential roles of incentive sensitization in both potentially problematic substance uses and behaviors.

## Introduction

Psychologists and neuroscientists have long strived to understand how addictions develop and what mechanisms maintain the usage, despite inevitable adverse effects. Many people use recreational drugs regularly, including alcohol, and for the vast majority, it does not raise serious concerns (1). Similarly, many people engage in potentially addictive behaviors without developing adverse consequences [e.g., (2)]. However, in some cases, casual use may result in compulsive behavior, which often persists even after the negative consequences predominate. The consequences of substance addictions are well-known, including medical problems, problems with employment, criminal behavior, and family relations (3). Also, both substance addictions and non-substance-related addictive behaviors negatively impact subjective wellbeing [e.g., (4, 5)].

The Incentive Sensitization Theory [IST (6); see also (7–10)] of addiction accounts for the psychological and neurobiological basis of drug craving, leading to substance use disorder and relapse. According to this theory, pleasure activates mechanisms of associative learning that normally functions to attribute incentive salience to reward cues, the process by which stimuli become “wanted.” Under normal circumstances, this mechanism is adaptive, promoting behavior to obtain fundamental rewards, such as food or sex (11). However, repeated and intermittent drug-taking behavior might lead to chronic neuroadaptational changes in the mesolimbic dopamine system, rendering this brain system hyper-sensitized to the drug and to the drug-associated cues, manifested in the increasing feeling of “want” toward the drug of abuse (12). Importantly, distinct neural structures (opioid, endocannabinoid, and GABA benzodiazepine neurotransmitter systems) are assumed to be responsible for the hedonic impact, i.e., “liking” of the substance, and they are not subject to sensitization (13), but to tolerance (6). Although the “wanting” and “liking” systems work in sync under normal circumstances, repeated drug taking can upset their balance—due to the different tendency to sensitization—resulting in increasing “wanting” with constant or reduced “liking.” This imbalance between the motivational and affective system is assumed to be responsible for the paradoxical situation, where the addict craves a substance, from which he/she does not expect pleasant feelings (9). The sensitized “wanting” system is then not specific to the subject of abuse, but spills over in a more general way, resulting in the “wanting” of multiple rewards (13), known as cross-sensitization; e.g., sensitization to a specific drug enhances the sensitivity to gambling-related stimuli (14). The sensitization progresses faster in the case of female rats, who show a more rapid and greater increase in motivation compared to males (15), which might contribute to the observation that women progress more rapidly from initial use to addiction (16). Importantly, the sensitization process is not restricted to substance abuse, but growing evidence suggests that it is involved in the development and maintenance of problematic behaviors, such as Internet-use disorder (17) or gambling, gaming, buying-shopping, and compulsive sexual behavior disorders (18).

Although most of the evidence for IST stems from studies on animals, more recently, the dissociation between “wanting” and “liking” has been examined in humans as well with contradictory results (19). This discrepancy may derive from the inconsistencies in the operationalization of the concepts of “wanting” and “liking.” While in animal experiments, the amount of work invested for reward (i.e. the number of lever presses) is a good indictator of “wanting”, and “liking” is well reflected in orofacial expressions (i.e. the rhythmic protrusion of the tongue) (13), human facial expressions are easily faked. Thus, the original research paradigm is not suitable to investigate the processes in question in humans (20). As Pool et al.'s (19) comprehensive review shows, there is no consensual paradigm investigating the IST in humans. From the 84 publications included in their review, 54% used physiological (e.g., mobilized effort, electromyography, food or drug administration), 31% used neurobiological (e.g., fMRI, PET, EEG, brain lesions), 11% used behavioral, and 5% used survey methods.

The low number of survey studies is due to the assumption that both “wanting” and “liking” work outside of conscious awareness—at least partly—(13), thus, explicit self-reports might not be suitable to differentiate between them. Robinson and Berridge (6) argue that humans may not be able to subjectively tell the difference between the two psychological processes of “wanting” vs. “liking,” and a person might mistake a change in incentive salience for a change in pleasure (“If I don't want it, then I must not like it”). Also, explicit incentive processes are relatively immune to manipulations of mesolimbic dopamine systems that change “wanting” (21). However, according to Berridge et al. (13), vivid imagery of reward cues can trigger measurable “wanting” properties; thus, an imagination situation might be sufficient to measure “wanting” and “liking” without the presence of the actual stimuli.

Despite the methodological concerns, a few survey methods were developed. Goldstein et al. (22) introduced the Sensitivity to Reinforcement of Addictive and other Primary Rewards (STRAP-R), which successfully differentiated between “wanting” and “liking” of the drug, food (22), and alcohol (23). The “strong desires to use” and “positive reinforcement from using” scales of Desires for Alcohol (DAQ) (24) and Speed Questionnaire (DSQ) (25) were used to measure alcohol- and amphetamine-related “wanting” and “liking” in the study by Willner et al. (26). They found that “wanting” and “liking” increased as a function of dependence on amphetamine or level of consumption in the case of alcohol, which partially supported IST.

An advantage of the questionnaire method is that it allows one to access larger samples and investigate the relationship of IST with other psychological constructs. For example, impulsivity is a significant marker for substance use disorders (27, 28) or non-substance-related potentially addictive behaviors, such as sexual behaviors [e.g., (29)], or Facebook use, or TV-series watching (30). However, the link between trait impulsivity and incentive sensitization has not yet been investigated. Impulsivity is a heterogeneous personality and behavioral construct (31), well described by the term “disinhibition,” referring to the not appropriate top-down control mechanisms supposed to suppress automatic or reward-driven responses (32). State and trait impulsivity can be distinguished. State impulsivity varies across time and is most often assessed *via* a neuropsychological test, while trait impulsivity is relatively constant and refers to an overall degree of impulsive behavior in an individual assessed by self-report questionnaires (33). According to the multidimensional UPPS-P model of trait impulsivity (34), five dimensions of impulsivity are distinct: *positive/negative urgency*, a tendency to act rashly in response to extreme positive/negative emotions, (lack of) *premeditation*, a tendency to consider the possible consequences of an act before engaging in it, *perseverance*, the ability to remain focused on a task, and *sensation seeking*, a tendency to enjoy and pursue stimulating activities (35). On the behavioral level, it is manifested in sudden actions in an unplanned manner to satisfy desires, such as acting on the spur of the moment or not considering the potential outcomes of an action before carrying it out (36). Impulsivity may manifest in maladaptive behaviors, such as aggressive or self-injuring behaviors, domestic violence, and suicide attempts (37). It is also associated with psychiatric disorders, such as antisocial personality disorder, borderline personality disorder, or bipolar disorder (38). In general, elevated trait impulsivity has been found in the patients with different substance use disorders, such as alcohol (39), cocaine (40), or tobacco (41). Also, problematic behaviors were linked to high impulsivity, such as problematic smartphone use (42), Internet-pornography-use disorder (43), problematic use of pornography (29), disordered use of social media (44), sexual addiction (45), cybersex addiction (46) or Internet addiction (47). The multidimensional approach (UPPS-P, see above) was suitable to examine more specific relationships between separate impulsivity-related constructs and addictions (48). Urgency was found to be an important predictor of the development of addictions. Positive urgency was associated with the quantity of alcohol students consumed on a single occasion and the negative outcomes experienced (49)— nicotine dependence and smoking status (50), while negative urgency was highly related to alcohol dependence (48), food addiction (51), and the severity of gambling disorder (52). The lack of perseverance also predicts the drinking quantity (48), smoking status (50), problematic use of pornography, binge eating, and use of drugs other than cannabis (53). However, it is important to note that impulsivity occurs on a continuum, thus, impulsivity *per se* is not an indicator of pathology (36). Another concept linked to various substance misuses is the reward deficiency syndrome (RDS), which is described as insufficiency of usual feelings of satisfaction, caused by a genetic defect (54). As natural rewards do not adequately stimulate the reward system of individuals with reward deficiency, they are at a greater risk of developing substance use disorder (55). Blum et al. (55) investigated the relationship between RDS and “wanting-liking,” and concluded that the two concepts are related. The mesolimbic dopamine dysregulation observed in RDS predisposes the individual to seek substances and behaviors, which is manifested in the “wanting” of those.

## Current Study

The aim of this study was to test the dissociation between “wanting” and “liking” across four substances (alcohol, nicotine, cannabis, other drugs) and ten potentially addictive behaviors (gambling, overeating, gaming, pornography use, sex, social media use, Internet use, TV-series watching, shopping, work). We hypothesized that “wanting” would increase with more frequent and intense use, while “liking” would stay steady or even decrease. We hypothesized that “wanting” of the substance/behavior which shows the greatest imbalance between the motivational and rewarding systems (i.e., the greatest difference between “wanting” and “liking”) would positively predict a general problem use construct, which involves all problem uses/behaviors specific to the individual. We hypothesized that “wanting” would be higher among women than men. Also, we hypothesized that impulsivity would positively predict “wanting” and the general problem use construct. We hypothesized that RDS would negatively predict “liking” and positively predict the general problem use construct. Finally, we hypothesized that indicators of problem uses/behaviors mediated by “wanting” would negatively predict wellbeing. The substances and behaviors of interest were based on Schulter et al.'s study (56), with the modification that cocaine was replaced by “other drugs,” and four additional behaviors were investigated (pornography use, social media use, TV-series watching, and Internet use), given the recent calls for further investigations [e.g., (18, 57, 58)].

## Methods

### Procedure and Participants

Data collection took place on social media sites and a popular Hungarian news portal *via* an online survey from June to September 2020. The study was advertised as a research project about the psychological factors of intense engagement in different behaviors. The survey completion took ~20–25 min. The study was conducted following the Declaration of Helsinki and was approved by the Joint Committee of Ethics of the Psychology Institutes, Hungary (Number 2020/258). The participants were informed about the aims of the study. Informed consent was obtained from the participants before data collection, and the participants were ensured of their anonymity. No personal information, that might have allowed identification, was asked, and a secure online platform (Qualtrics Research Suite; Qualtrics, Provo, UT) was used for data collection.

Overall, 749 participants (503 women, 67.2%) aged between 18 and 74 years (*M*__*ag*_e_ = 35.7 years, SD = 11.8) completed the questionnaire. As for the level of education, 16 participants had a primary level of education or less (2.1%), 46 had a vocational degree (6%), 105 had a high-school degree (13.8%), 553 had a college or university degree (72.8%), and 39 (5.1%) participants did not want to answer this question. Regarding the relationship status, 233 were single (30.7%), 463 were in any kind of romantic relationship (i.e., being in a romantic relationship or married) (61%), 25 chose the “other” option (3.2%), and 38 did not want to respond (5%).

### Measures

#### Screener for Substance and Behavioral Addictions

One item from the Screener for Substance and Behavioral Addictions (SSBA) (i.e., “I did it too much in the past 12 months.”) was used to measure potentially problematic behaviors. The participants were asked to indicate whether they did too much of any of the substances/behaviors of interest (alcohol, nicotine, cannabis, other drugs, gambling, overeating, gaming, pornography use, sex, social media use, Internet use, TV-series, shopping, work) in the past 12 months, with four answer options: Totally disagree, Partly disagree, Partly agree, and Totally agree. This measure has demonstrated good psychometric properties (56, 59) and allowed us to measure potentially addictive behaviors in a short time. Only substances/behaviors were assessed for “wanting,” “liking,” and frequency, for which “Partly agree” or “Totally agree” responses were indicated.

#### Imaginative “Wanting” and “Liking” Questionnaire

Imaginative “Wanting” and “Liking” Questionnaire (IWLQ) was designed to measure substance- and behavior-related “wanting” and “liking.” According to Berridge et al. (13), vivid imagery of reward cues may be sufficient to trigger measurable “wanting” properties, without the presence of the actual stimuli. Based on this, IWLQ contains micro scenarios in which subjects are asked to imagine themselves in certain substance- or behavior-related situations. For example: “Imagine yourself sitting in front of your favorite alcoholic drink, in the right time and place.” After the imagery call, participants have to indicate their (1) expected feelings on a ruler (−100: very bad, 0: neutral, 100: very good) before, during, and after use (e.g., “How would you feel before the first sip of your drink?,” “How would you feel during the drinking session?,” “How would you feel after the effects of the drinks are over?,” respectively). (2) The expected willpower they would need (0: nothing, 100: enormous) to resist/stop to participate in the behavior before, during, and after use (e.g., “How much willpower would you need in order to not drink from your drink?,” “How much willpower would you need in order to stop drinking after the first few sips?,” “How much willpower would you need in order to not drink in the next 24 h after you consumed the desired quantity?,” respectively). (3) Frequency of use (e.g., “How often do you consume alcohol?”. Answers: Weekly or less, 2–3 times a week, 4–5 times a week, every day, or nearly every day), and (4) intensity (for substances, e.g., “On days you consume alcohol, in general how much drinks do you have?”. Answers: 1, 2–3, 3–4, 5–6, 7, or more; for behaviors, e.g., “On days you overwork, in general how many extra hours?”. Answers: 1 hour or less, 2–3 h, 4–5 h, 6 h, or more).

We considered “willpower” as a good indicator of “wanting,” as it is the folk notion of self-control, the capacity to override one's impulses and habitual responses (60). The survey was created in a way that the IWLQ items of a substance/behavior were only shown to the participants if they previously indicated a substance or behavior as problematic (i.e., “Partly agree,” “Totally agree”).

#### Personal Well-Being Index—Adult

The Personal Well-Being Index—Adult (PWI-A) was used to measure subjective personal wellbeing (see the (61)). PWI-A measures seven domains of wellbeing—the standard of living, personal health, achieving in life, personal relationships, personal safety, community-connectedness, future security—and the participants indicated their answers on a ruler (0: not at all, 100: absolutely). The scale showed good reliability in the present sample (α = 0.82).

#### Barratt Impulsiveness Scale-Revised

Barratt Impulsiveness Scale-Revised (BIS-R-21) is a self-report measure designed to assess cognitive impulsivity, behavioral impulsivity, and impatience/restlessness (62). It includes 21 items (answer options: 1: *rarely/never*, 2: *occasionally*, 3: *often*, 4: *almost always/ always*). Since factor loadings of impatience/restlessness were low in our sample (mean 0.37 ± 0.09), and three items (items 2, 4, and 5) showed a floor effect (skewness >1), only cognitive impulsivity (e.g., “I plan tasks carefully”) and behavioral impulsivity (e.g., “I do things without thinking”) were included (14 items) in the present study. The scale showed good reliability in the present sample (α = 0.78).

#### Reward Deficiency Syndrome Questionnaire

Reward Deficiency Syndrome Questionnaire (RDSQ-29) was used to measure reward deficiency *via five* domains with 29 items: lack of sexual satisfaction (three items, e.g., “I can never get enough sex.”), activity (five items, e.g., “I cannot stand inactivity.”), social concerns (two items, e.g., “My friends and family often worry about my lifestyle.”), risk-seeking behavior (five items, e.g., “Extreme sports stimulate me.”), and additional items (14 items, e.g., “I like to be always active.”). The Participants indicated their answers using a four-point scale (1: *totally disagree*, 2: *partly disagree*, 3: *partly agree*, 4: *totally agree)* (63). The scale showed good reliability in the present sample (α = 0.91).

### Statistical Analyses

The 679 participants indicated a total of 2,770 uses/behaviors as problematic. For each problem behavior, weekly (F1) and daily (F2) frequency of activity and for each problem use, weekly frequency (F1) and quantity of substance per use (F2), were assessed. An intensity index was formed as the multiplication of F1 and F2. Separate multiple linear regression models were computed on standardized predictor and outcome variables in the three time points (before, during, and after) to analyze the relationship between “liking” or “wanting” and the intensity of the problem, as a function of problem type (behavior or substance use) and sex (female or male). Regression analyses were performed in R (4.0.2), package stats (64). Figures were plotted with ggplot2 (65).

Prior to the main analyses, we conducted separate factor analyses for RDS and BIS to ascertain their psychometric properties. Given the complexity of the hypothesized model, we opted to save these preliminary measurement models as factor scores and use them as input for the main analyses. Factor scores have the advantage over manifest scale scores of providing partial control for measurement error by allocating more weight to the items with lower error variances (66). The use of this approach is becoming increasingly popular (57, 67), further supporting our decision. For every participant, only one problem use/behavior was selected—the one with the highest difference between “wanting” and “liking” scores (76% behavioral). Normality was assessed by the investigation of skewness and kurtosis. Hae-Young Kim (68)—for sample sizes greater than 300—recommended the absolute values of 2.0 for skewness and 7.0 for kurtosis, which could be interpreted as thresholds for acceptability.

Next, structural equation modeling (SEM) with latent variables was conducted with diagonally weighted least squares estimation to examine the relationship pattern between impulsivity, reward deficiency, “wanting,” “liking,” wellbeing, and a general measure of problem use. The latent variable problem uses were inferred from the summed frequency (weekly use × regular amount of use) and summed subjective severity (SSBA responses) for all substances/behaviors indicated problematic, and the number of substances/behaviors indicated problematic (SSBA response partly agree/totally agree). Note, that while “wanting” and “liking” reflects one problem use—the one in which the difference between “wanting” and “liking” was the greatest, i.e., the most problematic according to IST—, the latent variable “problematic use” was constructed from all the substances/behaviors reported as problematic. SEM was performed in R (4.0.2), package Lavaan (69) with diagonally weighted least squares estimation. When assessing the models, multiple goodness-of-fit indices were observed (70) with good or acceptable values based on the following thresholds (71, 72). Regarding the comparative fit index (CFI) and Tucker–Lewis index (TLI), values higher than 0.95 indicated that a model had a good fit, whereas values higher than 0.90 indicated that a model had an acceptable fit. Regarding the root mean square error of approximation (RMSEA) with its 90% confidence interval (90% CI), a model can be considered good if its RMSEA value is below 0.06, whereas it can be considered acceptable if this value is below 0.08. In addition, following Schellenberg and Bailis's (73) suggestions, to examine the significance of indirect pathways in the mediation model, 95% bias-corrected bootstrapped confidence intervals (CIs) with 5,000 resamples were computed.

The missing values were replaced by a semi-random value, pooled from a set of numbers following the distribution of the particular variable.

## Results

**Figure 1:**
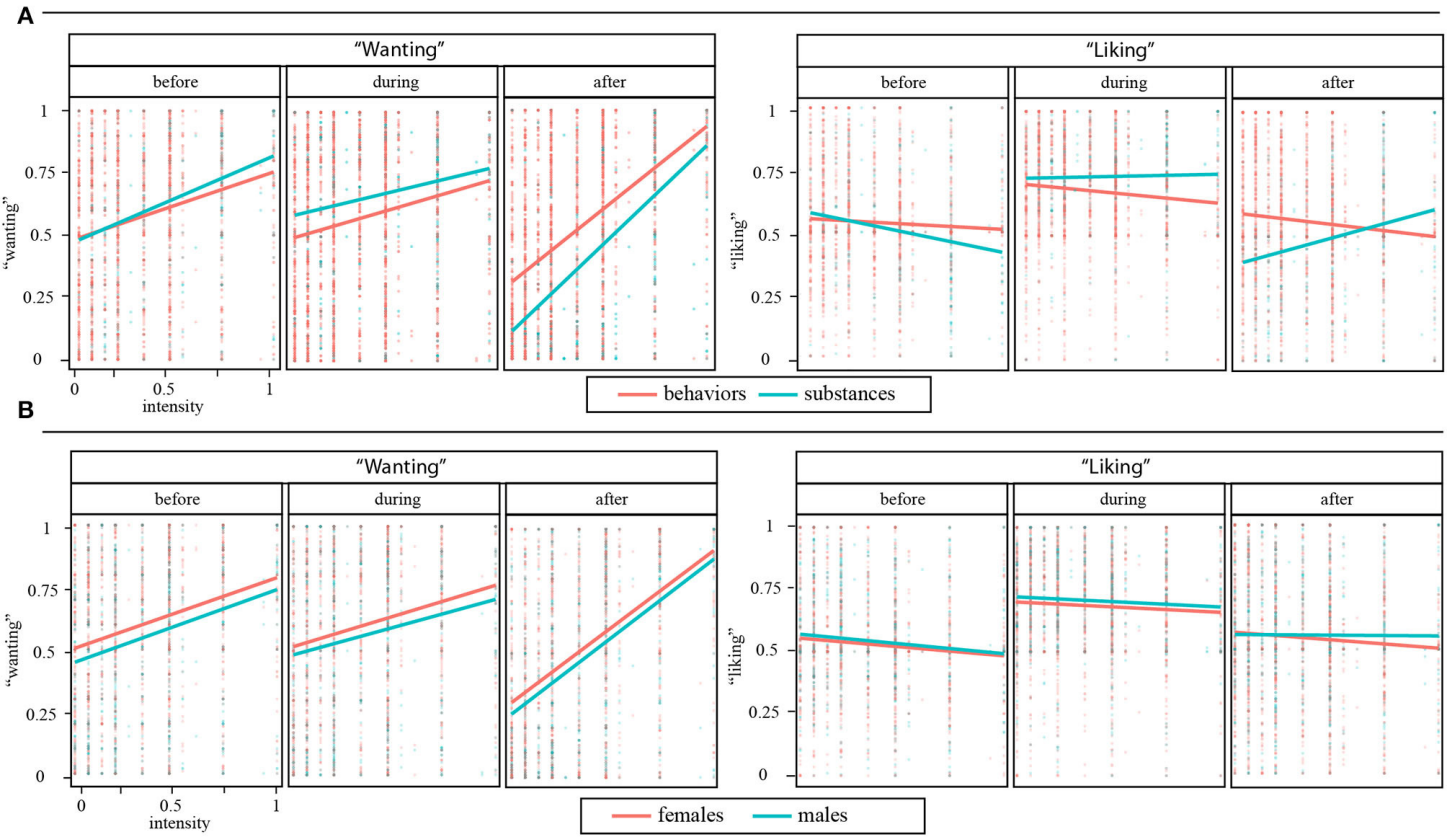
Scatter plots and linear regression lines of “wanting” and “liking” before, during, and after use/activity **(A)** difference between substance uses and behaviors, **(B)** difference between females and males. On the left side, the y-axis represents “wanting” (0: no willpower is needed to resist use/behavior; 1: enormous willpower is needed to resist use/behavior). On the right side, the y-axis represents “liking” (0: negative emotions; 0.5: neutral emotions; 1: positive emotions). The x-axis represents intensity (weekly frequency × daily frequency/amount of use).

Multiple linear regression was used to test if the intensity of usage (weekly frequency × daily frequency), problem type (substance, behavior), and sex (male, female), significantly predicted “wanting” in the three assessed time points (before, during, and after usage/activity) (see Table 1). The overall regressions were statistically significant. The explained variances were low in the case of before and during and moderate in the case of after use/activity. According to the predictions of IST, the intensity positively predicted “wanting” in all three time points, being the highest for “wanting-after.” In the case of problem behaviors, the slope coefficient of “wanting-after” was lower (see Figure 1A). According to the predictions of IST, “wanting” was lower in the male participants in all three time points and a Type × Sex interaction indicated, that “wanting-during” in the case of problem behaviors was even lower for the male participants (see Figure 1B).

**Table 1:** Relationship between “wanting,” ”liking,” and intensity as a function of problem type and sex.

	**“Wanting”**			**“Liking”**		
	**Before**	**During**	**After**	**Before**	**During**	**After**
F-value	F_(7, 2762)_ = 27.35	F_(7, 2762)_ = 20.37	F_(7, 2762)_ = 123.10	F_(7, 2762)_ = 6.90	F_(7, 2762)_ = 7.10	F_(7, 2762)_ = 15.33
*p*-value	*p* < 0.001	*p* < 0.001	*p* < 0.001	*p* < 0.001	*p* < 0.001	*p* < 0.001
Adjusted R2	0.06	0.05	0.24	0.01	0.02	0.03
Intensity	0.23 (0.02)^***^	0.18 (0.02)^***^	0.48 (0.20)^***^	−0.09 (0.02)^***^	−0.07 (0.02)^***^	–
Type	–	−0.09 (0.02)^***^	0.17 (0.02)^***^	–	−0.09(0.02)^***^	0.15 (0.02)^***^
Sex	−0.08 (0.02)^***^	−0.06 (0.02)^**^	−0.05 (0.02)^**^	–	0.04 (0.02)^*^	–
Intensity × Type	–	–	−0.03 (0.01)^*^	0.06 (0.02)^***^	−0.04 (0.02)^*^	−0.12 (0.02)^***^
Intensity × Sex	–	–	–	–	–	–
Type × Sex	–	−0.04 (0.02)^*^	–	–	–	–
Intensity × type × sex	–	–	–	–	–	0.06 (0.02)^**^

* *p < 0.05*;

** *p < 0.01*;

*** *p < 0.001*.

Multiple linear regressions were used to test the intensity of usage (weekly frequency × daily frequency), problem type (substance, behavior), and sex (male, female) (see Table 1). Although the explained varianve was low, Intensity negatively predicted “liking” before and during the use/activity. In the case of problem behaviors, for “liking-before” the slope coefficient was higher, while for “liking” during and after was lower compared to substances. The male subjects indicated higher “liking” during the uses/activities, and the Intensity × Type × Sex interaction indicated, that the male subjects indicated higher “liking” as a function of intensity in the case of problem behaviors.

**Figure 2:**
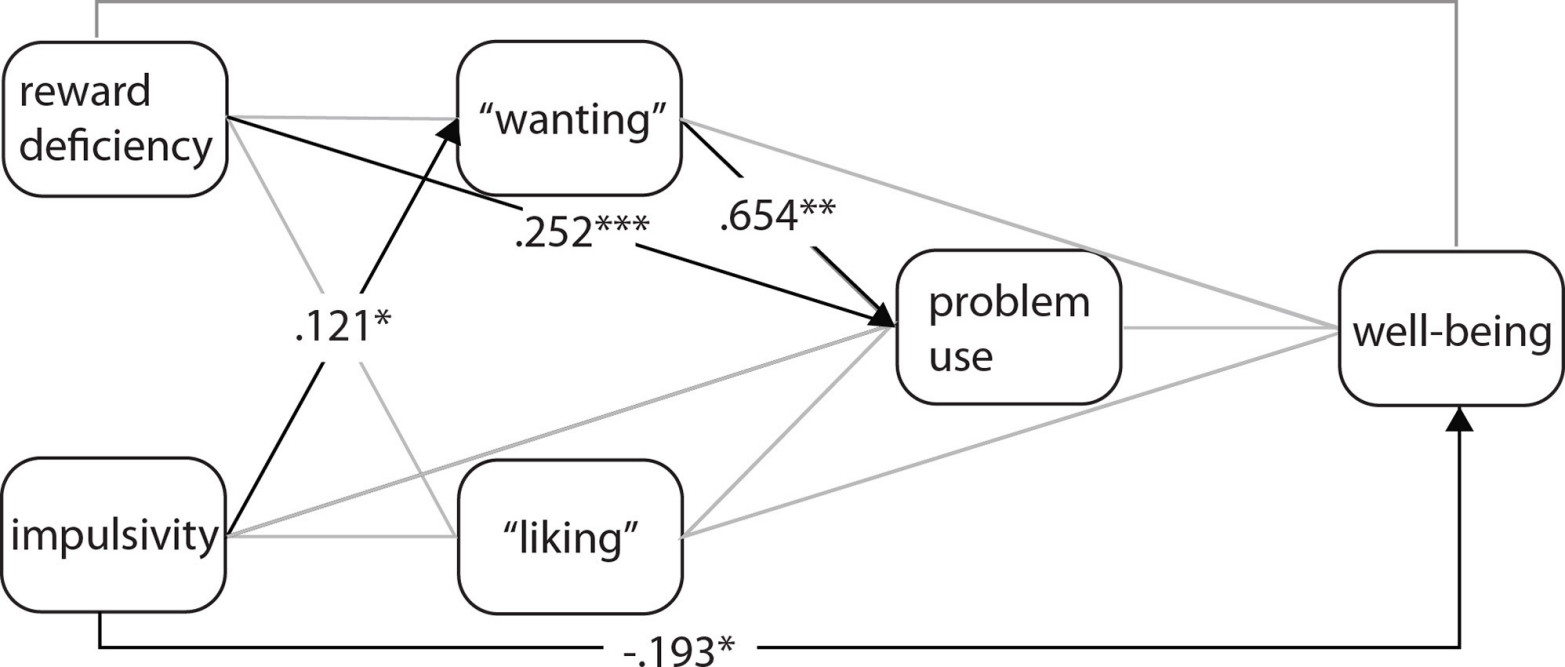
The role of impulsiveness (BIS), reward deficiency (RDS), “wanting” and “liking” on the wellbeing is mediated by problem use. The coefficients represent standardized regression weights. The gray arrows represent non-significant paths. *p < *0.05*, **p < *0.01*, *** *p* < *0.001*.

In the mediation model, the role of impulsiveness, reward deficiency, and problem uses was investigated regarding wellbeing through “wanting” and “liking” (Figure 2). Normality was examined and did not violate the thresholds of Kim (68), neither for skewness (ranging from −1.28 to 0.61), nor for kurtosis (ranging from 2.02 to 4.26). The model showed a good fit to the data (CFI: 0.964; TLI: 0.947; RMSEA: 0.047).

**Figure 3:**
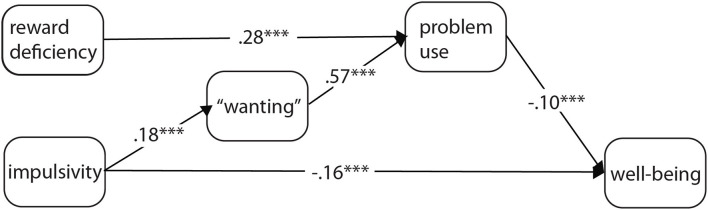
The final trimmed model of impulsivity, reward deficiency, “wanting,” problem uses, and wellbeing. The coefficients represent standardized regression weights. ***p < *0.001*.

The trimmed model also had a good fit to the data (CFI: 0.986; TLI: 0.980; RMSEA: 0.040), the results are reported in Table 2. The analyses showed that “wanting” was positively and weakly predicted by impulsivity, and “wanting” and reward deficiency had direct positive associations with problem uses (moderate and strong, respectively). Also, both problem uses and impulsivity negatively and weakly predicted wellbeing (Figure 3). Overall, the model explained 26% of the variance of problem uses, and 4% of wellbeing.

**Table 2:** Mediation analyses including direct and indirect effects for the trimmed model.

	**Direct effects**	
	**β**	**95% CI**
BIS → “wanting”	0.12 (*p* = 0.03)	(0.01, 0.23)
“Wanting” → problem uses	0.55 (*p* < 0.001)	(0.43, 0.69)
RDS → problem uses	0.24 (*p* < 0.001)	(0.14, 0.33)
Problem uses → wellbeing	−0.09 (*p* = 0.03)	(−0.16, −0.01)
BIS → wellbeing	−0.20 (*p* < 0.001)	(−0.29, −0.11)
	**Indirect effects**	
BIS → “wanting” → problem uses	0.07 (*p* = 0.04)	(0.01, 0.14)
BIS → “wanting” → problem uses → wellbeing	−0.099 (*p* = 0.048)	(−0.02, 0.00)
RDS → problem uses → wellbeing	−0.03 (*p* = 0.004)	(−0.05, −0.01)

*Bootstrapped confidence intervals were based on 5,000 replications and were estimated with diagonally weighted least squares. β = standardized regression weights, 95% CI bias-corrected bootstrapped confidence intervals*.

## Discussion

To date, relatively few studies have investigated the changes in substance- and behavior-related “wanting” and “liking” of individuals, especially in relation to other psychological factors. The aim of this study was to contribute to the scientific literature about IST by examining (1) the role of impulsivity, and reward deficiency as predictors of “wanting” and “liking,” and their associations with potentially problematic substance use/behaviors and wellbeing, and the (2) differences between “wanting” and “liking” as a function of usage frequency, severity, and sex differences.

Our first findings complement the prior studies in the field of IST studies showing that “wanting” increased with the self-reported severity, frequency, and intensity of potentially problematic use/behavior, while “liking” showed a slight decrease. These results support the accessibility of IST to individuals with potentially problematic substance use and behaviors in line with others [e.g., (12, 22, 23, 74–76)]. Also, the results suggest that the sensitization of the underlying processes of “wanting” is gradual (9), increasing with the frequency of usage.

Contrary to our expectations, self-estimated “wanting” followed by the use/behavior (i.e., “wanting-after”) showed the greatest increment as a function of usage intensity. Since “wanting” is linked to reward cues (77) triggering the urge to use (7), it is reasonable to expect the highest cue effect on “wanting-before.” A possible explanation is a methodological shortcoming that the question regarding “wanting-after” assessed the estimated willpower for abstinence of a well-defined time range (24 h), while no such time range was present before and during use/activity. It is possible that in the case of intense use/behavior, the presence of the time range amplified “wanting,” while no such effect was present for low/moderate intensity uses/behaviors, as reflected in low “wanting-after.” Another possible explanation is that moderate users are able to satisfy their “wanting” with use, but not intense users, supported by previous studies (12, 78) finding that implicit “wanting” scores of smokers were unaffected by nicotine deprivation (12 or 10 h of deprivation vs. immediately after smoke).

“Liking” slightly decreased as a function of usage intensity, but the explained variance was very low (1–3%), supporting that “liking” is largely unaffected by sensitization (7).

An important finding of the current study was that “wanting” showed similar tendencies to potentially problematic substance use and behaviors, which supports the applicability of IST to problematic behaviors, corroborating the findings of prior studies [e.g., (1, 79)]. Since the assessment of intensity for substance use (amount) and behaviors (time spent) was different, direct comparisons might be misleading and are out of the scope of the current study. Although the same restriction applies to the interpretation of “liking,” the tendency of “liking-after” for problem uses and problem behaviors was markedly different. Slightly positive effects were present for moderate behaviors, which decreased with intensity, being neutral for intense behaviors. In the case of substances, “liking-after” indicated slightly negative effects for moderate uses, which increased with intensity, being slightly positive for intense uses. We have no clear explanation for this unexpected pattern. A possible factor might be withdrawal relief [e.g., (80, 81)], but supplementary questions (e.g., about craving, guilt, relief, and euphoria) must be addressed to define the factors behind “liking,” preferably alongside substance-/behavior-specific questionnaires, to define the severity of the problem with higher accuracy and in more detail.

Sex differences in addiction research received increased attention in the past two decades (82), reporting that females generally exceed males in drug use (83) and problem behaviors, such as Internet addiction (84), overeating (85), gambling (86), or social media use (87). Expanding Kawa and Robinson's (15) findings in female rats, women reported a higher level of “wanting” in the present study, which was the most pronounced for problem behaviors, during the activity. This is in line with the findings that women showed increased reactivity to internal (emotional) and external (drug-associated) cues, leading to a higher propensity to drug relapse (88). Also, women reported slightly lower “liking” during the use/activity, which is in line with the previous studies indicating that women with addiction tend to display a higher level of negative emotions [e.g., (85, 89)]. As “wanting” defines the motivational aspects toward substances and behaviors, while decreased “liking” might be connected to coping with related motives, it is reasonable to assume a relationship with complex behaviors, contributing to other sex differences, such as vulnerability to substance use disorder (90–92), entering treatment (93), or motives of relapse (94, 95). Since this study is restricted regarding data from substance- and behavior-specific scales, more focused studies are needed.

An important finding of our study was that impulsivity positively predicted “wanting” of the substance/behavior, which showed the greatest imbalance between the motivational and rewarding systems (i.e., the greatest difference between “wanting” and “liking”). Although impulsivity is a significant marker for substance use disorders and problematic behaviors (27, 28), direct associations between impulsivity and “wanting” were not described in terms of IST. A transition from heightened impulsivity to heightened compulsivity might reflect the formation of problem behavior (29, 30, 96–98), as strong motivational urges can transform into automatic, compulsive actions (99). The I-PACE model (18), thus considers impulsivity as a general predisposing variable for addictive behaviors. Our results indicate that impulsivity might not be directly related to problem use but *via* the sensitization of the motivation system (“wanting”), resulting in compulsive behaviors. Previous studies indicated that high-trait impulsive individuals show higher cue reactivity for addiction-related cues [e.g., (100, 101)] and interaction between impulsivity and premorbid alternations in inhibitory processes were reported (102). This might explain the current results assuming that high-trait impulsivity increases cue reactivity, which triggers “wanting,” leading to decreased inhibitory and self-control abilities, which contributes to the development of problem substance use/behaviors. Considering that “wanting” and craving are closely related (103), this is in line with the findings of Meule and Blechert (104), who reported no direct effect of impulsivity on the body mass index, but higher impulsivity predicted more frequent and intense food cravings, which in turn predicted lower perceived self-regulatory success in eating, and that in turn predicted a higher body mass index. Also, in another study, higher rash impulsiveness (the tendency for approach despite potential negative consequences) did not directly predict the risk of relapse but was found to increase vulnerability to the craving, which in turn, increased the risk of relapse (105). However, the lack of direct association between impulsivity and problem behaviors is in contrast with a large literature [for a review see (106)], thus, more focused studies would be necessary to better understand the role of impulsivity-related constructs on incentive sensitization. Since “wanting” was derived from one behavior in our model, and problem use reflected all problematic behaviors, our results extend Berridge et al.'s (13) findings to humans that a sensitized dopamine system might not be specific to the subject of abuse, but spill over in a more general way. This observation might be potentially useful in understanding the mechanisms underlying the co-occurrence of substance addictions and problematic behaviors.

Corroborating previous findings [e.g., (54, 55)], reward deficiency positively predicted problem use in our study. According to the RDS hypothesis, individuals with higher levels of reward deficiency might be at greater risk of developing substance use disorder as natural rewards do not adequately stimulate their reward system (55). However, contrary to our expectations, reward deficiency did not predict “wanting” or “liking.” A potential explanation is that the positive effects related to short-term rewards might not be affected by reward deficiency, or even if they are, our tool was not able to capture it, since only subjective evaluation of the effects related to such rewards were assessed. However, this possible explanation warrants further investigation.

A great difficulty in human IST research is the operationalization of the “wanting” and “liking” concepts (107). In animal studies “wanting” is reflected by the work (e.g., paddle pushes) the animal is willing to invest in to get the substance (8). For people, determining the amount of work to be invested in would require serious calculations and in many cases (e.g., problematic behaviors such as social media use) would be difficult to interpret. Since cognitive wanting and liking are often used interchangeably (7), asking the person if she/he wants the substance might result in a “no” (although “wanting” might be high). For example, an individual recovering from alcohol-use problems might not want to drink alcohol at a social occasion, but still needs plenty of willpower to successfully resist. We argued that assessing “wanting” in an indirect way throughout the perceived cognitive resources of refusal might be a more accurate measure as it is not/less mixed with the affective aspects. For that reason, “wanting” was conceptualized in willpower, the folk notion of self-control, a conscious and effortful form of self-regulation, the capacity to override one's impulses, and automatic or habitual responses (i.e., “wanting”) (60). Since “wanting” and “liking” were assessed in an imagined ideal situation, it is assumable that the imagery triggered motivational/emotional signals (13) potentially lead to more accurate self-reports. Also, assessing an ideal scenario rather than at the given moment (i.e., “now”), might help to dissociate from the circumstances of the study participation (e.g., one might enjoy smoking cannabis after work, but not when completing the survey in the office). Also, the implicit nature of “wanting” and “liking” might be captured more adequately using a ruler for responses, and not categorical responses that may require a thoughtful elaboration of answers. Finally, in the IWLQ, “wanting” and “liking” were assessed in an imagined scenario before, during, and after use, allowing us to examine the dynamics of the two underlying systems over time. Yet, future studies are needed to examine the reliability and validity of the scale in diverse populations.

Several limitations warrant consideration. We used self-report scales in a self-selected sample that may introduce biases (e.g., overreporting or underreporting). Given the study's cross-sectional nature, causality cannot be inferred. This study did not test the dissociation explicitly across individuals with the clinical diagnosis of substance use or problematic engagement in a given behavior and non-problematic users. Rather, potentially problematic use was classified as “mild” and “severe” based on one item from the SSBA (56). To investigate the relationship between substance/behavioral addictions and the studied psychological constructs, further studies are needed, using substance- and behavior-specific scales. A weakness of the study is that we did not measure the duration/onset of the substance use/problem behavior. Such a variable would have allowed us to examine the sensitization process and the development of addictions in more detail. Also, from the 749 subjects, 70 did not indicate any substance/behavior as problematic, from whom we had no data on “wanting” and “liking.” In a future study, it would be beneficial to collect data from such participants to achieve a better understanding of the development of the early stages of problem behaviors and uses. Also, further studies should investigate the relation of positive (reward/pleasure) and negative (relief/satiation) reinforcement to “wanting” and “liking.” Considering that positive reinforcement may involve more elements of impulsivity, while negative reinforcement may involve more elements of compulsivity (108), it is reasonable to assume that such distinction would reveal important correlations between “wanting”-“liking” and impulsivity and reward deficiency. Also, since negative reinforcement can initiate and maintain self-medication behaviors (109), the dynamics of initial “wanting” and “liking” and their correlation to the motivation of usage should be investigated to test whether the willpower-based conceptualization of “wanting” is suitable to explain negative reinforcement-based motivational factors. Further, since “wanting” was conceptualized in the willpower to resist the use/activity, the effect of the underlying cause of restriction [i.e., internal (e.g., quitting attempt) or external (e.g., lack of resources)] should be investigated in the future, as it presumably has an effect on “liking.”

In sum, the present study largely supports the role of incentive sensitization in both potentially problematic substance use and behaviors. The results corroborate the notion that survey methods might be suitable for investigating IST. Moreover, the findings also suggest that impulsivity might not be directly associated with problematic engagement in substance use and different problematic behaviors (e.g., problematic pornography use) (29), but *via* “wanting” providing targets for future interventions.

## Data Availability Statement

The raw data supporting the conclusions of this article will be made available by the authors, without undue reservation.

## Ethics Statement

The studies involving human participants were reviewed and approved by ELTE PPK Ethical Committee. The patients/participants provided their written informed consent to participate in this study.

## Author Contributions

DF: conceptualization, methodology, visualization, writing—original draft, and formal analysis. BF: conceptualization, methodology, and writing—review and editing. ZD: conceptualization, funding acquisition, methodology, supervision, writing—review and editing. BB: conceptualization, methodology, formal analysis, writing—review and editing. All authors contributed to the article and approved the submitted version.

## Funding

The study was supported by the National Research, Development and Innovation Office (K126835, K131635, and PD138976). BB was supported by the Merit Scholarship Program for Foreign Students (PBEEE) awarded by the Ministère de l'Éducation et de l'Enseignement Supérieur (MEES) and by a postdoctoral fellowship from the SCOUP Team—Sexuality and Couples—Fonds de recherche du Québec, Société et Culture.

## Conflict of Interest

ELTE Eötvös Loránd University receives funding from the Szerencsejáték Ltd. to maintain a telephone helpline service for problematic gambling. ZD has also been involved in research on responsible gambling funded by Szerencsejáték Ltd. and the Gambling Supervision Board and provided educational materials for the Szerencsejáték Ltd's responsible gambling program.

## Publisher's Note

All claims expressed in this article are solely those of the authors and do not necessarily represent those of their affiliated organizations, or those of the publisher, the editors and the reviewers. Any product that may be evaluated in this article, or claim that may be made by its manufacturer, is not guaranteed or endorsed by the publisher.
